# Recording of diagnoses in public primary oral health care in a retrospective longitudinal observational study in a Finnish town: Underrepresentation of periodontitis diagnoses

**DOI:** 10.1002/cre2.291

**Published:** 2020-03-25

**Authors:** Jouko Kallio, Timo Kauppila, Lasse Suominen, Anna M. Heikkinen

**Affiliations:** ^1^ Administration of the Primary Care, City of Espoo Espoo Finland; ^2^ Department of General Practice and Primary Healthcare University of Helsinki Helsinki Finland; ^3^ Department of Oral and Maxillofacial Diseases, Institute of Dentistry University of Helsinki Helsinki Finland

**Keywords:** diagnosis, primary health care, quality improvement

## Abstract

**Objectives:**

This study investigates which oral diagnoses public primary dental care dentists record.

**Methods:**

An observational register‐based retrospective follow‐up study was performed in the public primary oral health care of a Finnish town after the dentists were advised to mark the diagnoses in their practices. The rate of recorded diagnoses resulting from visits to the public primary care dentists was studied. The assessed diagnoses were recorded with the 10th revision of the International Classification of Diseases. The distribution of diagnoses was recorded during a 2‐year follow‐up period.

**Results:**

The most frequent diagnosis groups were dental caries (K02, 38.6%), other diseases of dental hard tissues (K03, 14.9%), diseases of pulp and periapical tissues (K04, 11.4%), periodontal diseases (K05, 9.7%), and different types of bone fractures (S02, 8.1%). Periodontitis was underrepresented.

**Conclusions:**

In public primary oral health care, there may be difficulties in adequate recording of certain chronic diseases.

## 1 INTRODUCTION

Insufficient recording of diagnoses may hamper planning of health care and adequate allocation and management of resources (Fleming, Schellevis, & Paget, 2003), thus improving the recording of chronic diseases might theoretically serve as one of the first targets in improving the quality of care (Fleming et al., 2003; Hjerpe, Merlo, Ohlsson, Bengtsson Boström, & Lindblad, 2010). The frequent use of diagnostic terms for oral diseases by dentists should provide valuable data for management, and for targeting proper treatments of oral diseases, thus making primary oral health care more effective (Leake, 2002). Recording diagnoses might promote diagnostic thinking and thereby enhance rational judgment of treatment options which then may lead to better treatment outcomes and increased patient safety (Kalenderian et al., 2016). It might also facilitate the use of computer‐based clinical decision support systems (Kalenderian, Maramaldi, et al., 2016). Habitual recording of structured oral diseases diagnoses would allow for the aggregation and secondary analyses of clinical data to support downstream analyses for quality improvement and epidemiological assessments and provide a basis for reasonable incentive systems (Kalenderian et al., 2016). It could also support the formation of group practices by enhancing division of labor between dentists who specialize in different tasks and diseases (Obadan‐Udoh et al., 2017). Frequent recording of diagnoses also supports educational functions by providing the possibility to categorize patients in different treatment groups and thereby to compare the results of treatment actions. This will help to increase experience and expertise (Kalenderian et al., 2011).

For these reasons, the administration of the primary care of Espoo City considered the recording of diagnoses by both public primary care general practitioners (GPs) and dentists to be insufficient (Kallio, Kauppila, Suominen, & Heikkinen, 2017; Lehtovuori et al., 2018). In primary care, which thus was under the same administration, a financial incentive (group bonus) was used to enhance the recording of diagnoses by GPs (Lehtovuori et al., 2018). These financial incentives were found to be effective in increasing the overall recording of diagnoses from about 40 to 90% (Lehtovuori et al., 2018). By contrast, after paying special attention in 2009 to the recording of diagnoses for visits to public primary oral health care dentists, but without using incentives, the rate rose from 0 to 35% (Kallio et al., 2017).

Unexpectedly, group bonuses failed to enhance the recording of diagnoses of chronic diseases such as diabetes in the public primary care of Espoo (Lehtovuori et al., 2018). Such chronic diseases are the major cause of oral health problems (Kassebaum et al., 2017). The most common of such chronic oral diseases over the course of a lifetime is dental caries and periodontitis (Heilmann, Tsakos, & Watt, 2015).

The main aim of this present study was to investigate the range of diagnoses which were recorded to find out whether the data reflected the distribution of diagnoses in real clinical life in public primary oral health care and thus provided valid information about public health.

## 2 METHODS

The present work is a retrospective longitudinal observational study in the primary oral health care of the second largest city of Finland. This study was performed in the city of where in 2012 there were 254,000 inhabitants and 21 communal oral health care teams (also called cells). The number of dentists varied from 2 to 12 per team. There was also the same number of dental nurses (including dental hygienists) supporting the work of dentists in these teams. More detailed information about the functions and frequency of use in Espoo primary care at the time of this study has been described earlier (Kallio et al., 2017).

Recorded diagnoses as a percentage of all visits to communal primary care dentists in Espoo were the main measure of our study. Diagnoses were recorded by the dentists using the 10th revision of the International Classification of Diseases (ICD‐10, http://www.who.int/classifications/icd/en/HistoryOfICD.pdf). The data were obtained from the electronic Effica patient chart system (Tieto Ltd, Helsinki, Finland). This study was performed directly by computer from the patient register without identifying the patients. After creating an algorithm (by L. S.), the report generator of the Effica system provided the number of monthly visits to the dentists of the Espoo primary care, the number of those visits that had a recorded diagnosis and the diagnosis codes the dentists gave during these visits. We gathered the data retrospectively during 2010–2012. To study which diagnoses primary care dentists used, all diagnoses were recorded during the follow‐up.

No ethical approval was required because this study was made directly by computer from the patient register without identifying the patients or dentists in any way, (https://rekisteritutkimus.wordpress.com/luvat-ja-tietosuoja/). The register keeper (the health authorities of Espoo August 23, 2016) granted permission to carry out the study.

## 3 RESULTS

There were 102,895 visits with recorded diagnoses during 2010–2012, and 485 different diagnoses were used by the dentists of the public primary care. According to the reported distribution of diagnoses (Table 1), the most common diagnosis recorded by the dentists was dental caries (K02). The next most common diagnoses were other diseases of dental hard tissues (K03), diseases of pulp and periapical tissues (K04), and periodontal diseases (K05, Table 1).

**TABLE 1:** Percentage of diagnoses reported by the primary care dentists in main 10th revision of the International Classification of Diseases (ICD‐10) groups

ICD‐10	Diagnosis	%
K02	Dental caries	38.63
K03	Other diseases of hard tissues of teeth, excl. bruxism, dental caries, teeth‐grinding NOS	14.86
K04	Diseases of pulp and periapical tissues	11.42
K05	Gingivitis and periodontal diseases	9.65
S02	Fracture of skull and facial bones	8.07
Z01.2	Dental examination (without specific diagnose)	5.67
Z87.1	Personal history of diseases of the digestive system	3.34
K07	Dentofacial anomalies excl. hemifacial atrophy or hypertrophy, unilateral condylar hyperplasia or hypoplasia	2.38
K00	Disorders of tooth development and eruption	1.42
K08	Other disorders of teeth and supporting structures	0.98
F45.8	Other somatoform disorders (bruxism)	0.85
K01	Embedded and impacted teeth failing to erupt excl. K07.3	0.70
S03.2	Dislocation of tooth	0.31
K10	Other diseases of jaws	0.30
K06	Other disorders of gingiva and edentulous alveolar ridge	0.20
S00	Superficial injury of head	0.16
K13	Other diseases of lip and oral mucosa	0.12
S01	Open wound of head	0.12
K12	Stomatitis and related lesions	0.11
K11	Diseases of salivary glands	0.09
Z71.1	Person with feared complaint in whom no diagnosis is made	0.07
Z97.2	Presence of dental prosthetic device	0.07
G47.3	Sleep apnoea	0.05
L43.9	Lichen planus	0.05
K14	Diseases of tongue	0.05

About 6% of the patients were examined for putative oral diseases without any specific diagnosis. Somatoform disorders such as bruxism (F45.8) were relatively rare. Of individual oral diagnoses, dental caries was clearly the most prominent one. Pulpitis and caries of enamel were the next most common oral diseases (Table 2).

**TABLE 2:** Specific oral diseases diagnosed by primary care dentists (according to 10th revision of the International Classification of Diseases [ICD‐10] system)

ICD‐10	Diagnosis	%
K02.1	Caries of dentine	33.73
K03.64	Deposits (accretions) on teeth (supragingival calculus)	9.43
S02.51	Fracture of the crown of the tooth (without contact to the pulp)	6.26
Z01.2	Dental examination (without specific diagnose)	5.67
K04.0	Pulpitis	4.93
Z87.1	Personal history of diseases of the digestive system	3.43
K02.0	Caries limited to enamel	3.07
K04.5	Chronic apical periodontitis	2.54
K03.65	Deposits (accretions) on teeth (subgingival calculus)	2.37
K04.1	Necrosis of pulp	1.81
K05.10	Chronic gingivitis	1.80
K05.31	Chronic periodontitis (complicated parodontitis)	1.54
K05.30	Chronic periodontitis (uncomplicated parodontitis)	1.34
K02.8	Other dental caries	0.92
S02.54	Fracture of tooth	0.89
F45.8	Somastoform disorders (bruxism)	0.85
K03.66	Deposits (accretions) on teeth, plaque	0.71
K07.25	Crossbite	0.69
K08.3	Retained dental root	0.69
K04.7	Periapical abscess without sinus	0.54
K05.4	Periodontosis	0.54
K03.80	Other specified diseases of hard tissues of teeth (sensitive dentine)	0.53
K05.38	Other chronic parodontitis	0.49
K03.1	Abrasion of teeth	0.48
K05.18	Other chronic gingivitis	0.46
K02.2	Caries of cementum	0.43
K07.2	Anomalies of dental arch relationship	0.41
K04.6	Periapical abscess with sinus	0.39
K00.68	Other disturbance in tooth eruption	0.37
K02.9	Dental caries, unspecified	0.36
K07.60	Temporomandibular joint disorders	0.34
S02.52	Fracture of tooth crown reaching pulpa	0.34
K07.30	Spacing, abnormal of tooth or teeth	0.34
K00.7	Teething syndrome	0.29
K00.40	Enamel hypoplasia	0.29
K01.17	Erupted or only partially erupted tooth because of obstruction by another tooth	0.29
K05.39	Nonspecific chronic parodontitis	0.28
K05.19	Nonspecific chronic gingivitis	0.28
K10.3	Alveolitis of jaws	0.27
K03.22	Erosion of teeth (other)	0.26
K03.0	Excessive attrition of teeth	0.25
S02.59	Nonspecified fracture of tooth	0.23
S03.20	Dislocation of tooth	0.23
K08.80	Toothache (without specific diagnosis)	0.21
K01.16	Erupted or only partially erupted tooth	0.19
S02.53	Fracture of root of tooth	0.19
K03.29	Erosion of teeth (unknown reason)	0.19
K05.09	Acute gingivitis	0.18
K05.20	Parodontal abscess without sinus	0.15
K02.3	Arrested dental caries	0.12

**FIGURE 1:**
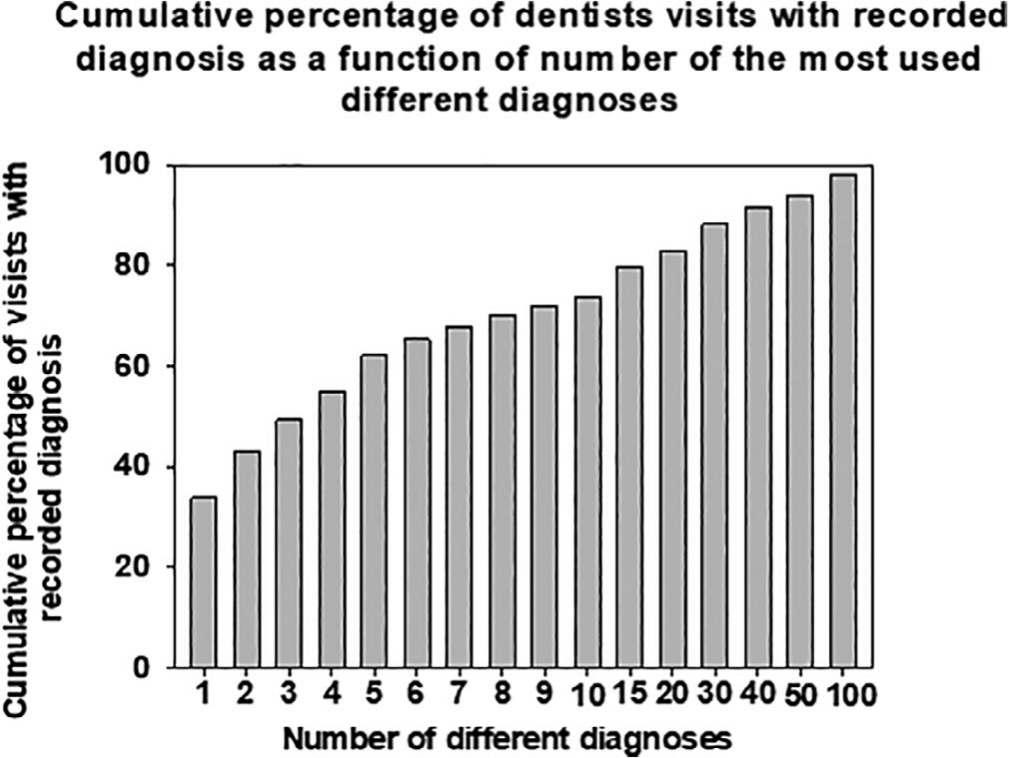
Cumulative coverage of visits to primary care dentists as a function of the most used diagnose

Only one diagnosis (Caries of dentine, K02.1) was recorded in more than 25% of the cases in the study. The three most frequently used diagnoses accounted for approximately 50% of cases (Figure 1). The 10 most frequently used diagnoses were recorded in about 75% of the cases. The 40 most frequently used diagnoses accounted for 90% of cases.

## 4 DISCUSSION

To the best of our knowledge, there are no former reports about the distribution of diagnoses which were recorded by public primary oral health care dentists. About 60% of the diagnostic terms entered to the electronic patient chart concerned dental caries, other diseases of dental hard tissue or diseases of pulp and periapical tissue. Caries was recorded in about 40% of the patients but periodontitis was recorded in only 14% of cases. About one patient in 20 had no disease detected by public primary care dentists. It thus appears that 90% of a primary care dentist's work can be described with the 40 most used diagnoses.

The percentage of caries diagnosis in the study was about 40% of all diagnoses. Although data were collected from the population of only one town in Finland, this figure is in line with Finnish observational studies (Suominen et al., 2018) and international studies (Marcenes et al., 2013). Thus, the recording of caries diagnoses by primary care dentists was at an adequate level.

The situation was different with periodontitis. According to a Finnish clinical cohort study based on clinical examination of patients, about 50–60% of the patients suffered from signs of periodontitis (Suominen et al., 2018). Similar figures have been reported in a clinical study from the United States, which was similarly performed by examining a sample of patients (Eke et al., 2015). Analogously, according to epidemiologic surveys directed to dentists, periodontitis is a major common oral disease among adults in Finland and over 60% of the Finnish population suffers from it (Suominen‐Taipale, Nordblad, Vehkalahti, & Aromaa, 2008). However, less than 10% of ICD‐10 terms included codes related to periodontal diseases in the present study. This figure is near the prevalence of severe periodontitis, which is related to the apparent risk of loss of teeth (Marcenes et al., 2013). A putative explanation for this discrepancy may be found from a recent report from a neighboring country, Sweden, which has a relatively similar primary dental care system to the Finnish. Based on clinical registers, Swedish researchers observed that periodontologic status was accorded to only about 20–40% of the patients visiting primary care dentists (von Bültzingslöwen et al., 2019). Either communal dentists fail to diagnose these diseases, they do not record these diagnoses despite observing their presence, or they record only severe cases. In the worst case scenario, periodontitis is ignored.

It is also possible that the public primary care dentists do not record periodontitis under the correct terms or that they do not record it at all. There are former studies suggesting that factors related to the use of the applied diagnostic terminology itself, or to the use of the terminology as part of clinic workflow and the related use of the electronic patient chart interface may influence the frequency of recording diagnoses and the quality of these recordings in oral health care (Obadan‐Udoh et al., 2017). There may also be aspects, such as extra work required to learn to use novel, possibly changing terminology, financial incentives, and fear of loss of autonomy which may decrease enthusiasm to record diagnoses (Ramoni et al., 2017; Tokede et al., 2013; Walji et al., 2013). In addition, cultural traditions (e.g., instead of recording diagnoses dentists are used to recording treatments and procedures) may have an influence on these factors (Ramoni et al., 2017; Walji et al., 2013). Nevertheless, the low level of recording periodontitis may hamper the reliability of register‐based studies that are solely based on entries on patient charts instead of direct patient examination.

The main strength of the present study was the completeness of the data. Every visit was included in the study. The computerized patient chart system reached every public primary care dentist in the city of Espoo. However, the accuracy of all the diagnoses cannot be guaranteed in the present study. There are differences in how individual GPs or dentists code their diagnoses. However, the data were so large that differences in coding between different GPs or dentists are likely to vanish in random deviation. Our data give no information as to how adequate the recorded diagnoses were or to which measures, if any, they led. Lack of data about individual dentists and their patients is another major flaw of this study. The lack of these data inhibits us from drawing conclusions about the demography of the patients or about variability in the behavior of dentists in making diagnoses.

## 5 CONCLUSIONS

Commitment to the idea that the recording of diagnoses is beneficial does not guarantee that all oral health diagnoses are recorded properly. There is a high level of variability in the quality of recording diagnoses of oral diseases in the public primary care.

## CONFLICTS OF INTEREST

The authors declare no potential conflict of interest.

## AUTHOR CONTRIBUTIONS

Conceptualization: Jouko Kallio, Timo Kauppila, and Anna M. Heikkinen; methodology: Jouko Kallio, Timo Kauppila, and Lasse Suominen; software: Jouko Kallio and Lasse Suominen; validation: Jouko Kallio and Timo Kauppila; formal analysis: Timo Kauppila and Anna M. Heikkinen; investigation: Timo Kauppila and Anna M. Heikkinen; resources: Jouko Kallio and Lasse Suominen; data curation: Lasse Suominen; writing—original draft preparation: Timo Kauppila; writing—review and editing: Jouko Kallio, Timo Kauppila, and Anna M. Heikkinen; supervision: Jouko Kallio and Lasse Suominen; project administration, Jouko Kallio; funding acquisition: Jouko Kallio and Lasse Suominen.
